# Factors associated with psychological distress of workers in the Finnish Evangelical Lutheran Church

**DOI:** 10.1186/s12889-024-18165-x

**Published:** 2024-03-21

**Authors:** Tuire Kuusi, Kati Tervo-Niemelä, Satu Viertiö

**Affiliations:** 1 University of the Arts, Helsinki, Finland; 2 University of Eastern Finland, Joensuu, Finland; 3 The Department of Public Health and Welfare, The Finnish Institute for Health and Welfare, Helsinki, Finland

**Keywords:** Mental health, Psychological distress, Workload, Pastor, Church musician, Diaconal worker, Youth worker

## Abstract

**Background:**

The work of church employees contains many elements causing symptoms of stress and anxiety. They can lead into psychological distress and possibly indicate the beginning of a more serious psychological state. Women seem to be more disposed to psychological stress than men. We investigated factors contributing to psychological distress among women and men in four professions of the Evangelical Lutheran Church of Finland (ELCF).

**Methods:**

A link to an electronic survey was sent to the members of respective trade unions of four professions of the ELCF, and we got responses from pastors (*n* = 241), church musicians (*n* = 92), diaconal workers (*n* = 85) and youth workers (*n* = 56). Psychological distress was assessed using the Mental Health Inventory-5 (MHI-5; cut-off value ≤ 52 indicating severe distress). We used logistic regression to examine sociodemographic, health-related, and work-related factors that could potentially be associated with psychological distress.

**Results:**

We found severe psychological distress in all profession groups. Gender differences were scarce. Loneliness was the most important factor associated with psychological distress in both men and women (OR 14.01; 95% CI 2.68–73.25 and OR 7.84; 3.44–17.88, respectively), and among pastors and church musicians (OR 8.10; 2.83–23.16 and OR 24.36; 2.78–213.72, respectively). High mental strain of work was associated with distress in women (OR 2.45; 1.01–5.97). Good work satisfaction was a protective factor for men and women (OR 0.06; 95% CI 0.01–0.40 and OR 0.61; 0.18–0.40, respectively) and for pastors and church musicians (OR 0.22; 0.08–0.73 and OR 0.06; 0.01–0.43, respectively). For women, additional protective factors were being a pastor (OR 0.26; 0.07–0.95), or youth worker (OR 0.08; 0.01–0.48), and good self-reported health (OR 0.38; 0.18–0.82).

**Conclusion:**

Even though we found some protective factors, the share of workers with severe distress was higher in all profession groups of the ELCF than in the general population. Loneliness was the strongest stressor among both genders and high mental strain among women. The result may reflect unconscious mental strain or subordination to the prevailing working conditions. More attention should be paid to the mental wellbeing and work conditions of church employees.

## Background

Pastors’ and other church employees’ work contains a great deal of factors that may be connected with psychological distress [1–3]. Previous studies, mostly conducted among the clergy, show several features that church workers find burdensome in their work. These include long and unpredictable working hours, the feeling of being constantly on-call, an increasing amount of bureaucracy, work overload, role ambiguity, difficulties in combining private and working life, and unsatisfactory financial compensation [3–12].

In this article, we are focusing on psychological distress among church workers in the Evangelical Lutheran Church of Finland (ELCF). Previous studies have shown high distress measures for church workers in the ELCF. Even though these studies did not use similar measures for workload, exhaustion and psychological distress, the tendency can be read from the results: approximately 50% of the diaconal workers experienced that their work was distressing [13], and more than 70% of them reported having too much work and difficulties in defining the responsibilities of their work [14], while approximately one fifth of both pastors and church musicians had similar experiences [15]. The share of youth workers who felt exhausted was 15% [16]. A recent study [17] on the effects of the COVID-19 pandemic revealed that nearly two-thirds of the workers in the church experienced that psychological distress, workload and exhaustion had increased during and after the pandemic, and this was especially the case with those in diaconal work (85%) and youth work (78%), and also with pastors (60%) and church musicians (50%).

### Psychological distress

Psychological distress is commonly related to non-specific symptoms of stress, anxiety, and depression [18], and it can be measured with a five-item Mental Health Inventory (MHI-5) [19, 20]. These five items measure how often a respondent has felt nervous, sad, and depressed as opposed to feeling calm, happy, and peaceful (for details, see *Variables*). Previous studies show that several factors can contribute to increasing risk of psychological distress. These include low level of job satisfaction [21], lack of social and emotional support [22], emotional and social loneliness [23], and harmful lifestyles such as alcohol use [24], smoking [25], and substance use [26].

Previous studies in Western countries have shown that women are more disposed to psychological stress, depression, and anxiety than men [20, 27–29]. The risk factors affecting the gender difference can be biological, psychological, and social [30, 31]. Expectations for women and men can be different, as can the societal roles. Earlier study [32] has also shown that work engagement is gendered, and it is easier for men to experience the main components of engagement (psychological meaningfulness, safety, and availability) at work. Further, work-family conflict can increase mental stress, and – especially during parenting – it is more common among women than men, although the gender gap in Europe is small [33, 34]. Work-family conflict has recently been studied with physicians and shown to be associated with depression, burnout, and emotional exhaustion. The study showed that there was an increase in gender inequalities during the COVID-19 pandemic; women increased their responsibility for home and children with the consequence that they experienced more work-to-family and family-to work conflict, as well as depressive and anxiety symptoms, than men [35].

### Reward and distress of vocational work

The church work involves meeting people and supporting them in different life situations. Enthusiasm and joy are often emphasized in vocational professions such as the professions at church [36]. Research has shown that almost all Finnish church workers enjoy their work and consider their work meaningful and useful, and in general, work-related wellbeing factors are high in most workplaces [37]. Psychological distress can, however, be common in vocational work with people, although such work may also be considered particularly meaningful [38]. Church workers are among those professions to whom people turn to when seeking professional help. Church workers are expected to be able to help those who are facing traumatic life situations, death, illness, or sorrow, that is, the workers need abilities to handle emotional distress of the parish members. If there are also other work demands (e.g. workload, quick pace of work), the emotional demands may increase the worker’s distress [38]. Some professions have no regular working hours, and this may cause distress because of the conflict between the service mission, needs of the parish members, and the worker’s need to rest and recovery. Finally, recent studies show a notable decrease in work-related wellbeing among pastors and church musicians of the ELCF, during the COVID-19 pandemic [39].

In this study we examined four profession groups at the ELCF. Their duties and education differ from each other, but there also are important common aspects. First, the task of the workers within each group are the same regardless of the gender. Second, the workers in all groups have higher education either at Master of Arts or Bachelor of Arts (sometimes even doctoral) level. The four groups, their duties and education are as follows:

**Pastor** is a ministry of the church, and the officials are ordained as pastors. The special tasks of pastors are to provide public service and share sacred sacraments, to perform other church services, and to act in pastoral care. Their work is not tied to predetermined, regular working hours but spread over the week and days. In addition to their theological expertise, pastors need a wide range of interaction skills and presence in their daily work with individuals, groups, and communities. At the same time, they need the ability to structure and relate their work to the strategy, objectives, and activities of the church [40]. The master’s level education of the pastors takes place at theological faculties of universities. In Finland women have been ordained as pastors for 30 years, and currently women make up almost half of the acting pastors in the ELCF. However, there are less women in leadership positions: only 20% of the vicars are women. There is still a small part of the parish members (5%) and employees (9%) who do not accept women as pastors [41].

The **church musicians** of the ELCF run the musical life of their parish and work with parishioners of all ages. Their work consists of musical activities in services and various activities in the parish, e.g., choir conducting, music group conducting, music education at confirmation schools and children’s music classes, etc. Like with pastors, also church musicians’ work is without predetermined regular working hours and spread over the week and days. In addition to their musical expertise, they also need theological knowledge, since pastoral duties and meeting the parishioners in various major life events is part of their work [42]. Church musicians have a music-specific degree which can be either a master’s level degree from a university or a bachelor’s level degree (most often) from a university of applied sciences [43]. Women have been able to work as church musicians in Finland for more than 60 years, and currently approximately 67% of all church musicians in Finland are female [17].

**Diaconal work** is also part of the basic spiritual work of the ELCF. The diaconal workers are educated in universities of applied sciences. There are two options: *BA of social services, diaconal work* or *BA of health care, diaconal nursing*. The latter also gives the student the professional qualifications of a nurse. Most diaconal workers have regular working hours. The work involves people’s everyday lives and dealing with complex issues in a creative and professional manner. Diaconal workers assist and support individuals, families, groups, and communities in various life circumstances and help them to cope independently [44]. Of all diaconal workers in Finland, approximately 92% are female.

**Christian Youth Work** is based on the mission of the Church, and the workers are supposed to be committed to the church’s basic spiritual mission and values [45]. As with the diaconal workers, the youth workers are also educated in universities of applied science. Their BA can be the *BA of social services*, further defined as Christian Youth Work, or *Bachelor of Humanities* specializing in the youth work of the church. Youth workers are professionals who support young people’s social and emotional growth, and they work with children, young people, and families in various settings. The work includes planning, developing, and implementing activities together with children, young people, and their families. Most often the work has regular working hours, but it can also include camps and excursions [46]. The share of female youth workers in Finland is approximately 66%.

## Aim

The study aimed at investigating factors contributing to psychological distress among workers in the ELCF. Our study has a special focus on comparing four professions in the church (pastors, church musicians, diaconal workers, and youth workers) and examining gender differences within the professions: how the church workers in different profession groups experience psychological distress and are there differences between male and female workers. Since we focus on four profession groups, each with different work descriptions, we can assume that the analyses would reveal differences in experiences of distress both between profession groups and between men and women workers within each profession group.

## Materials and methods

### Data collection

For data collection we used a questionnaire (see Variables). We contacted the participants via the trade unions of the pastors, church musicians, diaconal workers, and youth workers of the ELCF at the end of 2021. In these profession groups, a vast majority of church workers are members of these trade unions, which means that most of these workers can be reached via these unions. The unions informed all their members about the study by e-mail and sent the link to the questionnaire via the same e-mail. We also informed about the research on social media (Facebook) for contacting those who possibly had not read their e-mail or had ignored it. However, we have no data about the number of workers who were reached via these channels. The total number of respondents was 537, and they were divided as follows: 258 pastors, 100 church musicians, 88 diaconal workers, 61 youth workers, and 30 others (they did not belong to any of the above-mentioned groups and were excluded from our analyses). For the pastors, the number of respondents accounts for approximately 13% of the members (1978 in total) of the clergy union. For church musicians, the share is approximately 15% (100 out of the total of 658), while among diaconal workers (total 1168) and youth workers (total 769) the shares are lower (7.5% and 7.9%, respectively). Based on gender and diocese division, the respondents well represented the four groups of workers of the ELCF.

### Variables

The questionnaire was created for the present study on the basis of various sources. We selected various quantitative measures for examining the participants’ health and wellbeing, work, and their possibilities to combine work and family responsibilities from the *Health, Wellbeing and Service Use Study* by the Finnish Institute of Health and Welfare (see, e.g. the questionnaire from 2017 [47]. Psychological distress was assessed in this study using the Mental Health Inventory (MHI-5) based on the SF-36 questionnaire [19]. It is a self-report instrument to measure health-related quality of life and to detect anxiety and depression symptoms. The MHI-5 consists of five questions: ‘How much of the time during the last month have you: 1) been a very nervous person, 2) felt downhearted and blue, 3) felt calm and peaceful, 4) felt so down in the dumps that nothing could cheer you up, and 5) been a happy person?’ The five possible responses to the questions were scored between 1 and 5. Items 3 and 5 ask about positive feelings and their scoring was done in reverse. All scores were then converted to fit a range from 0 to 100, with low scores indicating more psychological distress. To measure clinically significant psychological distress, we used the cut-off of 52 points derived from the Eurobarometer survey in 2002 [48]. Cronbach’s alpha for MHI-5 was α = 0.885; which indicates good internal consistency reliability.

The age of the participants was divided into three equal-sized categories: under 46 years, 46–55 years, and 56 years or more. Marital status was categorized as married/cohabiting, divorced/widowed, and single. Living alone was categorized based on a response of yes or no. Subjectively experienced loneliness was divided into two categories: never/seldom/sometimes and quite often/all the time. Self-reported health was asked using the following question: ‘How would you describe your current state of health?’ and it was categorized as good/rather good, moderate, and rather poor/poor.

Alcohol consumption was assessed using the Alcohol Use Disorders Identification Test (AUDIT-C) [49]. It consists of three questions, each scored 0–4: ‘How often do you have a drink containing alcohol?’ (alternatives: never, once a month or less often, 2–4 time a month, 2–3 times a week, 4 or more times a week), ‘How many standard drinks of alcohol do you have on a typical day when you are drinking?’ (alternatives: 1–2, 3–4, 5–6, 7–9, 10 or more), ‘How often do you have 5 or more drinks on one occasion?’ (never, less than once a month, once a month, once a week, daily or almost daily). A total score of six or more for men and five or more for women indicates at-risk drinking. Cigarette smoking was asked: ‘Do you smoke at the moment (cigarettes, cigars, or pipe)?’ and it was divided into three categories: never, sometimes/daily, and ‘I have stopped smoking’.

Potential work-family conflict was assessed using the following question: ‘Are the following statements about home and work accurate for you?’ The respondents were asked to agree or disagree with two statements: ‘I feel I am neglecting home issues because of my work’ and ‘I often find it difficult to concentrate on my work because of home issues’. The answers were divided into two categories: completely/fairly accurate and completely/fairly inaccurate.

Types of employment contracts were studied with two variables. One was whether the worker had full-time or part-time work and the other was whether the worker had permanent or fixed-term work. Job satisfaction was measured using the following question: ‘How satisfied are you with your present work?’ The responses were divided into three categories: extremely/fairly satisfied, neither satisfied nor dissatisfied, and fairly/extremely dissatisfied. Mental and physical strain of work was measured using the following question: ‘What is/was your most recent job like (physically and mentally)?’ Answers were divided into three categories as follows: low strain (light or fairly light), moderate strain (a bit strenuous), and high strain (quite or very strenuous). In regression analyses, mental and physical strain were divided into two categories: low strain and moderate/high strain. Questions related to job satisfaction, job strain, and work-family conflict are from the Finnish Quality of Work Life Surveys 1977–2008 [50].

### Data analysis

Analyses were conducted using the SAS Enterprise Guide 7.1 [51]. We analysed the distribution of sociodemographic variables, health-related variables, and work-related variables (below, work and non-work variables) in four profession groups for both genders. Differences in the categorical variables were tested using the Chi square test. We also analysed the psychological distress measured with MHI-5 (yes/no; cut-off value of MHI-5 ≤ 52 indicating severe distress) in the four profession groups.

We used logistic regression analysis to examine associations between the work and non-work variables, and psychological distress in the whole study sample, separately in men and women. Dependent variable in the model was psychological distress (MHI-5) and independent variables were included in the model simultaneously.

In the result section, the results are organized so that we first present the differences in the four profession groups and between genders and, second, present the results concerning work and non-work variables explaining psychological distress. Finally, we present the association between the same variables and psychological distress in two profession groups, pastors and church musicians, individually. Due to the relatively small sample size of diaconal workers and youth workers, it was not expedient to analyse these profession groups separately.

## Results

The results for MHI-5 (Table 1) show that the shares of ELCF workers who experienced clinically significant psychological distress were high. For women, the shares varied between 14.7% (Christian youth workers) and 22.5% (church musicians), and for men they varied between 18.6% (pastors) and 38.1% (church musicians). Even though the results in all profession groups showed that men experience clinically significant psychological distress more often than women, the differences are not statistically significant in any profession group. In other words, the results show that work at ELCF causes psychological distress, but the finding is not specific for any profession.

We continued by analysing gender differences in non-work variables but, generally, we did not find statistically significant differences in most of the factors. These include loneliness, suicide thoughts or self-reported physical health, and marital status. There were no statistically significant gender differences in at-risk drinking either. The only statistically significant difference was in smoking: male church musicians were smoking more often than female. Generally, the church workers seemed to be in rather good physical health: more than 60% of participants in all groups self-reported good or rather good physical health.

When focusing on the work variables, we found only few statistically significant differences between men and women: Female diaconal workers reported more often than men that they felt they were neglecting home issues because of their work. Male pastors, on the other hand, reported more often than female that it was difficult to concentrate on the work because of home issues. The smallest difference between the genders in work-family conflict was in church musicians.

**Table 1:** Descriptive information of the study sample by profession groups and gender. Statistical significance of the differences between men and women

Gender	Pastors			Church musicians			Diaconal workers			Christian youth workers		
	Men (n = 80)	Women (n = 161)	P-value^1^	Men (n = 21)	Women (n = 71)	P-value^1^	Men (n = 5)^2^	Women (n = 80)	P-value^1^	Men (n = 22)	Women (n = 34)	P-value^1^
	n (%)	n (%)		n (%)	n (%)			n (%)		n (%)	n (%)	
**MHI-5 (cut-off ≤ 52 points = psychological distress)**	15 (18.8)	27 (16.8)	0.7028	8 (38.1)	16 (22.5)	0.1537		14 (17.5)	0.3061	5 (22.7)	4 (14.7)	0.4440
**MHI-5 mean (95% CI)**	69.8	69.9		58.9	66.9			67.6		68.5	68.6	
**Age (years)**			0.9246			0.7100			0.6701			0.1272
under 46 46–55 56 or older **Age (mean)**	27 (32.5) 20 (24.1) 36 (43.4)	55 (34.0) 41 (25.3) 66 (40.7)		4 (18.2) 9 (40.9) 9 (40.9)	19 (26.8) 27 (38.0) 25 (35.2)			23 (28.8) 25 (31.2) 32 (40.0)		8 (36.4) 5 (22.7) 9 (40.9)	22 (62.9) 6 (17.1) 7 (20.0)	
**Marital status**			0.0517			0.6325			0.4987			0.1303
Married/cohabiting	66 (80.5)	104 (65.4)		15 (68.2)	54 (77.1)			61 (76.3)		15 (68.2)	27 (77.1)	
Separated/divorced/widowed	6 (7.3)	20 (12.6)		2 (9.1)	6 (8.6)			12 (15.0)		4 (18.2)	1 (2.9)	
Single	10 (12.2)	35 (22.0)		5 (22.7)	10 (14.3)			7 (8.7)		3 (13.6)	7 (20.0)	
**Lives alone**	17 (20.5)	40 (24.8)	0.4454	6 (27.3)	15 (21.1)	0.5469		11 (13.9)	0.7065	7 (31.8)	6 (17.1)	0.1986
**Loneliness**												
Never/seldom/sometimes Quite often/all the time	73 (88.0) 10 (12.0)	138 (85.2) 24 (14.8)	0.5533	16 (72.7) 6 (27.3)	57 (80.3) 14 (19.7)	0.4511		74 (92.5) 6 (7.5)	0.3239	18 (81.8) 4 (18.2)	28 (80.0) 7 (20.0)	0.8655
**Suicide thoughts over the past 12 months**	6 (7.2)	14 (8.6)	0.7022	2 (9.1)	2 (2.8)	0.2050		4 (5.0)	0.1667	1 (4.6)	3 (8.6)	0.5624
**Self-reported current physical health**												
Good/rather good Moderate Rather poor/poor	57 (68.7) 20 (24.1) 6 (7.2)	111 (68.5) 39 (24.1) 12 (7.4)	0.9987	15 (68.2) 5 (22.7) 2 (9.1)	48 (67.6) 12 (16.9) 11 (15.5)	0.6686		54 (67.5) 19 (23.8) 7 (8.7)	0.7507	15 (68.2) 6 (27.3) 1 (4.6)	21 (60.0) 12 (34.3) 2 (5.7)	0.8234
**At-risk drinking (AUDIT-C)** ^**3**^	9 (10.8)	28 (17.3)	0.1827	5 (22.7)	10 (14.1)	0.3355		8 (10.0)	0.4575	3 (13.6)	9 (25.7)	0.2762
**Smoking**												
Never Sometimes or daily Quit	53 (63.9) 14 (16.9) 16 (19.3)	112 (69.6) 23 (14.3) 26 (16.2)	0.6650	12 (57.1) 5 (23.8) 4 (19.1)	61 (85.9) 4 (5.6) 6 (8.5)	**0.0120**		59 (73.8) 3 (3.8) 18 (22.5)	0.8934	10 (50.0) 3 (15.0) 7 (3.0)	19 (54.2) 8 (22.9) 8 (22.9)	0.5697
**Full-time work**	78 (94.0)	143 (88.3)	0.1916	17 (77.3)	64 (90.1)	0.1157		73 (91.3)	0.4899	19 (86.4)	31 (91.2)	0.5696
**Permanent work**	65 (81.3)	127 (81.9)	0.8975	20 (95.2)	66 (97.1)	0.6861		71 (89.9)	0.4888	21 (100)	30 (85.7)	0.0695
**The effect of COVID pandemic on work load**												
No effect Work load increased Work load decreased	27 (32.5) 43 (51.8) 13 (15.7)	34 (21.1) 100 (62.1) 27 (16.8)	0.1433	8 (36.4) 11 (50.0) 3 (13.6)	17 (23.9) 33 (46.5) 21 (29.6)	0.2643		9 (11.3) 68 (85.0) 3 (3.7)	0.7758	1 (5.3) 17 (89.5) 1 (5.3)	5 (14.3) 26 (74.3) 4 (11.4)	0.4636
**Mental strain of work**									0.1181			0.7736
Low Moderate High	6 (7.2) 20 (24.1) 57 (68.7	9 (5.6) 42 (25.9) 111 (68.4)	0.8505	5 (22.7) 67 (31.8) 10 (45.5)	9 (12.7) 22 (31.0) 40 (56.3)	0.4721		2 (2.5) 16 (20.0) 62 (75.5)		1 (5.3) 4 (21.1) 14 (73.7)	2 (5.7) 9 (25.7) 24 (68.8)	
**Physical strain of work**												
Low Moderate High	75 (90.4) 7 (8.4) 1 (1.2)	135 (83.3) 25 (15.4) 2 (1.3)	0.3050	16 (72.7) 46(27.3) 0	45 (64.3) 18 (25.7) 7 (10.0)	0.3019		64 (80.0) 14 (17.5) 2 (2.5)	0.9316	15 (68.2) 6 (27.3) 1 (4.5)	22 (62.9) 12 (34.3) 1 (2.8)	0.8273
**Work satisfaction**												
Extremely/fairly satisfied Neither satisfied nor dissatisfied Fairly/extremely dissatisfied	59 (71.1) 16 (19.3) 8 (9.6)	126 (78.3) 15 (9.3) 20 (12.4)	0.0813	12 (63.2) 1 (5.3) 6 (31.6)	50 (71.4) 8 (11.4) 12 (17.2)	0.2589		63 (78.8) 13 (16.3) 4 (5.0)	0.8645	17 (77.3) 2 (9.1) 3 (13.6)	22 (62.9) 9 (25.7) 4 (11.4)	0.3015
**Family-work conflict**												
I feel I am neglecting home issues because of my work I often find it difficult to concentrate on my work because of domestic issues	40 (50.6) 15 (18.8)	96 (60.8) 15 (9.7)	0.1373 **0.0483**	13 (61.9) 3 (14.3)	48 (69.6) 11 (16.9)	0.5107 0.7759		53 (66.3) 8 (10.5)	**0.0030** 0.4448	13 (61.9) 2 (9.1)	21 (61.8) 8 (24.2)	0.9917 0.1535

^1^ P-values indicating the statistical significance between men and women lower than .05 (statistically significant) are in bold print

^2^ Not reported due to small N

^3^ At-risk drinking is assessed as AUDIT-C, men ≥ 6 points and women ≥ 5 points

### Associations between psychological distress (MHI-5) and work and non-work variables

When we searched for factors potentially influencing psychological distress in the whole data (Table 2), we found the strongest association in loneliness, in both men and women. On the other hand, good work satisfaction was associated with less psychological distress in both men and women. Differences between the genders were few: Within women, the profession of pastor and youth worker was a protective factor. Health-related factors were associated with distress only in women, as self-reported health was a protective factor. Moderate or high mental strain of work was associated with distress in women.

**Table 2:** Logistic regression analysis (odds ratios with 95% confidence intervals) of non-work and work variables with psychological distress (MHI-5 cut-off ≤ 52 points) in the study sample. Variables were included in the model simultaneously.

	Men (n = 134)		Women (n = 323)	
	OR (95% CI)^2^	P value^3^	OR (95% CI)^2^	P value ^3^
**Sociodemographic factors**				
Profession ^1^				
Pastor Church musician Diaconal worker Youth worker	0.40 (0.05–3.21) 0.52 (0.04–6.77) 0.15 (0.01–3.06) 0.27 (0.02–3.43)	0.3850 0.6176 0.2196 0.3156	**0.26 (0.07–0.95)** 0.26 (0.06–1.12) 0.38 (0.10–1.45) **0.08 (0.01–0.48)**	**0.0416** 0.0702 0.1564 **0.0055**
Age (years)				
under 45 46–55 56 or older	1 0.19 (0.03–1.14) 0.47 (1.11–2.02)	0.0702 0.3085	1 0.59 (0.25–1.42) 0.45 (0.18–1.09)	0.2419 0.0774
Marital status				
Married/cohabiting	1			
Divorced/separated/widowed	0.95 (0.14–6.67)	0.9579	1.20 (0.44–3.29)	0.7215
Single	0.25 (0.04–1.44)	0.1203	2.02 (0.74–5.50)	0.1706
**Health-related factors**				
Self-reported good physical health	0.75 (0.20–2.84)	0.6761	**0.38 (0.18–0.82)**	**0.0132**
At-risk drinking	2.60 (0.35–19.43)	0.3525	0.64 (0.20–2.03)	0.4463
Smoking				
Never Sometimes or daily Quit	1 3.29 (0.70–15.40) 2.36 (0.58–9.62)	0.1318 0.2323	1 1.30 (0.34–4.95) 2.14 (0.92–5.02)	0.6975 0.0790
Lonely quite often or all the time	**14.01 (2.68–73.25)**	**0.0018**	**7.84 (3.44–17.88)**	**< .0001**
**Work-related factors**				
Part-time work	0.93 (0.06–13.11)	0.9577	0.40 (0.10–1.57)	0.1885
Fixed-term work	0.78 (0.15–4.04)	0.7850	0.78 (0.28–2.20)	0.6382
Work satisfaction				
Extremely/fairly satisfied Neither satisfied nor dissatisfied Fairly/extremely dissatisfied	**0.06 (0.01–0.40)** 0.18 (0.02–1.36) 1	**0.0039** 0.0955	**0.16 (0.06–0.47)** 0.61 (0.18–2.11)	**0.0008** 0.4339
Mental strain of work Low Moderate or high	1 0.99 (0.25–3.95)	0.9922	**2.45 (1.01–5.97)**	**0.0481**
Physical strain of work Low Moderate or high	1 0.96 (0.22–4.20)	0.9521	0.99 (0.44–2.24)	0.9821
I feel I am neglecting home issues because of my work ^4^	2.21 (0.56–8.68)	0.2574	2.02 (0.88–4.66)	0.0982
I often find it difficult to concentrate on my work because of domestic issues ^4^	0.41 (0.08–2.24)	0.3049	1.96 (0.75–5.14)	0.1705

^1^ The reference group of each profession group are those who are not in that profession

^2^ OR = Odds ratio, 95% CI = 95% confidence interval. Bold ratios: statistically significant results

^3^ P-values lower than 0.05 (statistically significant) are in bold print

^4^ N indicates the number of yes answers

In Table 3 we show the results of multivariable logistic regression analyses separately for two of the profession groups, namely pastors and church musicians. In both groups, loneliness was associated with the largest risk of psychological distress, and good work satisfaction was a protective factor against distress. In pastors, self-reported good health was also associated with less distress.

**Table 3:** Logistic regression analysis (odds ratios with 95% confidence intervals) of non-work and work variables with psychological distress (MHI-5 cut-off ≤52 points) in two profession groups. Variables were included in the model simultaneously

	Pastors (N = 241)		Church musicians (N = 92)	
	OR (95% CI)^1^	P value^2^	OR (95% CI)^1^	P value^2^
**Sociodemographic factors**				
Gender				
Male Female	1.13 (0.42–2.99) 1	0.8097	1.21 (0.17–8.71) 1	0.8479
Age (years)				
under 45 46–55 56 or older	1 0.80 (0.27–2.38) 0.43 (0.15–1.27)	0.6873 0.1272	1 1.03 (0.15–7.12) 1.76 (0.25–12.25)	0.9733 0.5682
Marital status				
Married/cohabiting Divorced/separated/widowed Single	1 2.39 (0.71–7.98) 1.50 (0.42–5.42)	0.1558 0.5346	1 0.22 (0.01–4.79) 0.08 (0.01–1.32)	0.4282 0.0774
**Health-related factors**				
Self-reported good health	**0.29 (0.12–0.73)**	**0.0087**	2.72 (0.30–24.50)	0.3723
At-risk drinking	0.57 (0.14–2.32))	0.4300	0.30 (0.02–5.35)	0.4160
Smoking				
Never Sometimes of daily Quit	1 0.68 (0.17–2.72) 0.63 (0.18–2.19)	0.5830 0.4638	1 0.60 (0.03–13.40) 0.70 (0.06–8.71)	0.7485 0.7817
Lonely quite often or all the time	**8.10 (2.83–23.16)**	**< 0.0001**	**24.36 (2.78–213.72)**	**0.0040**
**Work-related factors**				
Part-time work	1.18 (0.21–6.65)	0.8504	11.28 (0.71–178.18)	0.0854
Fixed-term work	0.85 (0.25–2.83)	0.7859	1.05 (0.10–11.14)	0.9687
Work satisfaction				
Extremely/fairly satisfied Neither satisfied nor dissatisfied Fairly/extremely dissatisfied	**0.22 (0.08–0.73)** 0.67 (0.15–2.96) 1	**0.0133** 0.5973	**0.06 (0.01–0.43)** 0.15 (0.01–1.99) 1	**0.0058** 0.1090
Mental strain of work				
Low Moderate or high	1 1.37 (0.50–3.77)	0.5443	1 1.33 (0.28–6.40)	0.7217
Physical strain of work Low Moderate or high	1 0.83 (0.23–2.95)	0.7722	1 1.89 (0.36–9.82)	0.4483
I feel I am neglecting home issues because of my work ^3^	1.21 (0.46–3.21)	0.6952	4.51 (0.55–37.28)	0.1621
I often find it difficult to concentrate on my work because of domestic issues ^3^	0.51 (0.13–1.97)	0.3259	4.11 (0.34–50.17)	0.2686

^1^ OR = Odds ratio, 95% CI = 95% confidence interval. Bold ratios: statistically significant results

^2^ P-values lower than 0.05 (statistically significant) are in bold print

^3^ N indicates the number of yes answers

## Discussion

In this cross-sectional study of four Finnish church professions, we found clearly higher psychological distress for the ELCF workers than in the general population, both for women and men. Yet, we found few work and wellbeing factors that were associated with psychological distress. Furthermore, we found some differences between the four profession groups we studied. Some differences between genders were also found in regression analysis.

In a recent population-based study about the working population in Finland, there were 8.8% of male and 11% of female respondents who self-reported psychological distress at a level that is clinically significant and would need to be treated [20]. Compared to the working population in general, male church musicians, who were most stressed, reported almost four and a half times more distress and female church musicians reported twice as much.

Heavy alcohol intake has been found to be associated with an increased risk for, e.g., lower mental health and depressive symptoms [26, 52]. Our results showed that at-risk drinking was rarer within the Church workers than in the Finnish population with the same educational degree and age range [53]: the percentage of at-risk drinking in the general population was 35.6% for men and 18.2% for women, while in our sample it was between 10.8% and 22.7% for men and between 10% and 25.7% for women. Only female Christian youth workers had a higher rate (25.7%) than women in the general population. Earlier research has shown that there is a connection between low-level alcohol use and religiousness, especially in rural areas [54], and that deaths from alcohol-related diseases were especially rare in Finnish church musicians, both male and female [55]. We did not find association between psychological distress and alcohol use. Therefore, alcohol consumption is not the reason for high psychological distress in our sample.

Smoking was rarer in our sample than in previous study of the Finnish general population [20]. Women were smoking less than men, but those women who had quit smoking had more stress than others. Current smoking has been found to be associated with non-specific psychological distress [56]. We do not know how long ago the participants had quit smoking, but it is widely recognized that psychological distress is common while quitting smoking [57].

Common work conditions that have been found to cause distress at work are, for example, job insecurity [58], shift work [59], and physical exposure to harmful working conditions [60]. Job insecurity is associated with part-time and temporary work contracts [61]. In our sample, the largest share of part-time employment contracts was with male church musicians (22.7%), who also reported most distress. Further, in our sample the share of respondents with temporary contracts was clearly lower (between 2.9% for church musicians and 18.7% for pastors) than at the church in general (varying between 18.6% for church musicians to 22.6% for pastors) [62], indicating that those who might have most distress because of the temporary work did not even respond. This may explain why temporary contracts were not associated with distress in the two profession groups we studied in logistic regression analysis, namely, pastors and church musicians.

The church’s professions could be compared to shift work due to non-standard working hours. In particular, pastors and church musicians also work during weekends, their working hours are irregular, and it is not always easy for them to take their weekly days off. Further, during the pandemic, church workers reported increased amount of work, new duties, and changes in working environment, and they also reported that work became more demanding during the pandemic [17, 39]. These factors can explain the greater amount of distress in the church employees of our study compared to the general working population [20, 38].

Job strain is high in working conditions in which the workers have too little control over their work, demands are too high, and they get little social support in the workplace [63]. Church workers in Finland reported remarkably more mental job strain than participants in the general population [20]. In our study sample, high mental strain was associated with psychological distress in women, but not in men. This is opposite to a previous study [20], where mental strain of work was associated with psychological distress in men. Generally, vocational work is characterized by enthusiasm and joy [36], and mental strain of the work may provide meaning and quality in work [38]. Yet, in these respects, church work is the same for men and women. Earlier study has, further, shown that women had clearly higher anxiety during the pandemic [64] and that women are less resilient and have a higher prevalence of mood and psychological disorders after disasters [65]. Even though the pandemic cannot be compared to a disaster, it can be called an abnormal situation causing mental strain.

Education of the workers in the ELCF is quite high, as bachelor’s or master’s degree is needed for all the studied professions: pastors, cantors, youth workers and diaconal workers. Higher education has been shown to correlate with good physical and mental health [66]. More than 60% of the participants reported their physical health as good or rather good, which compares well with the physical health status of the general population [67]. In our study self-reported good health was associated with less distress in pastors and in women.

Our study showed that 59% of pastors and 47% of church musicians reported that COVID-19 pandemic had increased their workload. The increase in workload was very close to ours in a previous study (approximately 59% of pastors and 49% of church musicians reported so in [39]). It is important to remember that some workers experienced a decrease in workload (17% of pastors and 26% of church musicians in our study compared to 26% and 33% in [39]).

In our study the share of those who were extremely or fairly satisfied with work varied between 62.9% (Christian youth worker women) and 78.8% (diaconal worker women). Even the highest percentage was lower than it was in Finland 2018 (88%; [68]), most likely reflecting the above-mentioned changes during the COVID-19 pandemic (see also [17]). Previous longitudinal research among pastors and church musicians also shows that work satisfaction among both professions had decreased during the COVID-19 pandemic and the share of those feeling burdened in their work had increased [69]. In an earlier meta-analysis [21], as in our study, psychological distress was more common in workers with low work satisfaction. The same meta-analysis found that burnout was the most important mediator between work dissatisfaction and mental health problems. If work dissatisfaction causes burnout, it may, if untreated, lead to mental health problems.

Loneliness was the most significant factor in psychological distress in our study. Loneliness is associated with both physical health problems [70] and mental health disorders [23, 71]. In our study sample the association of loneliness with distress was almost two times bigger in men than in women, but in a previous population-based study [20] the association was equal, and generally women report loneliness more commonly than men [72]. For men, loneliness was the only risk factor for distress in our study, while for women the risk factors also included earlier smoking and high mental strain of work. These findings reflect that loneliness was a greater factor in wellbeing for men than for women in our study sample.

Our results show that the church workers had good physical health, low alcohol consumption and relatively high work satisfaction; these all are sources of psychological wellbeing. Commitment, higher age, and higher educational status can also be factors contributing to the wellbeing of the church workers via increasing work engagement regardless of the specific profession. In general, we did not find major gender differences within the professions, nor did we find major differences between the professions. This could be interpreted to indicate that there is equity in work tasks and working conditions within the Finnish church. This is important, since, as stated, there still are members of the church who do not accept women as pastors [41]. Another possible explanation is that women use the questionnaire scales differently from men: women do not choose ‘daily’ or ‘all the time’ options to describe their distress or bad feelings, since they do not find them exceptional, only normal [73].

### Strengths and limitations

There have been numerous studies related to pastors’ work wellbeing. However, there are few studies conducted among other professions in the church. In this study we have studied psychological distress among church musicians, diaconal workers, and youth workers in addition to pastors. In this study, the special focus was on health and wellbeing on the one hand, and gender on the other. The study was conducted during the COVID-19 pandemic. Even though there are also other studies of church workers both during the COVID-19 pandemic [39] and earlier (e.g., [68]), those studies used different measures for examining well-being. Taken together, the study provides valuable information on the wellbeing among four professions of the ELCF during a special time period. However, this special time period also needs to be kept in mind when reading the results.

The limitations of the study are as follows. (1) We collected the data during the COVID-19 pandemic, and, hence, the data does not include experiences before or after the pandemic. (2) Because the pre-pandemic studies used different measures of well-being, we were rarely able to compare their results to those of our study. (3) The number of respondents was relatively low. Approximately 16% of church musicians who were members of the union, responded; of pastors, the share was 13%, and it was even lower for diaconal workers and Christian youth workers. Due to small sample, we could not report the male diaconal workers’ data. (4) It is possible that the attrition of data was selective and that we got responses from those who were specifically interested in the topic. (5) The number of respondents with temporary contracts was low, indicating that one cause of distress was missing in our data. Yet, as stated, the respondents represented well all dioceses of the ELCF as well as the gender division of the profession groups.

## Conclusions

The share of church workers, both men and women and in all four professions, who experienced clinically significant psychological distress (measured by the MHI-5) was higher than in the general population. The results might be indicative of unconscious mental strain or subordination to the prevailing working conditions, especially since the workers had reported excessive work and unclear working conditions already before the COVID-19 pandemic, and these have further increased during the pandemic. We did not find major differences between the professions, nor between the genders, indicating equity in work tasks and working conditions in the ELCF. We call for further research into the elements of distress (as measured by the MHI-5) and experiences of loneliness with church workers in particular and vocational work in general. More attention should be paid to the mental wellbeing of church employees in the workplace, and the workers should be heard more about their working conditions. If mental strain goes unnoticed and is continuously neglected, it may lead to more serious mental ill health.

## Data Availability

The data will be saved for five years after the publication of the study, after which they are archived in the Finnish Social Science Data Archive.
